# Biofoundries are a nucleating hub for industrial translation

**DOI:** 10.1093/synbio/ysab013

**Published:** 2021-06-23

**Authors:** Tabasum Farzaneh, Paul S Freemont

**Affiliations:** UK Innovation and Knowledge Centre for Synthetic Biology (SynbiCITE) and the London Biofoundry, Imperial College Translation & Innovation Hub, London, UK; Section of Structural and Synthetic Biology, Department of Infectious Disease, Imperial College London, London, UK; UK Innovation and Knowledge Centre for Synthetic Biology (SynbiCITE) and the London Biofoundry, Imperial College Translation & Innovation Hub, London, UK; Section of Structural and Synthetic Biology, Department of Infectious Disease, Imperial College London, London, UK; UK Dementia Research Institute Care Research and Technology Centre, Imperial College London, London, UK

**Keywords:** biofoundries, innovation, standardization, automation, commercialization

## Abstract

Contemporary synthetic biology embraces the entire innovation pipeline; it is a transformative technology platform impacting new applications and improving existing industrial products and processes. However, challenges still emerge at the interface of upstream and downstream processes, integral to the value chain. It is now clear that biofoundries have a key role to play in addressing this; they provide unique and accessible infrastructure to drive the standardization necessary to deliver systematic design and engineering of biological systems and workflows. As for other biofoundries, the success of the London Biofoundry has been in part due to its expertise in establishing channels for industrial translation through its extensive strategic collaborations. It has also become cemented as a key component of various consortia and partnerships that serve the broader bioeconomy and industrial strategies. Adopting a networked approach enables links to be made between infrastructure, researchers, industrialists and policy makers to de-risk the economic challenges of scale-up, as well as contribute to the growing bioeconomy.

Synthetic biology (also referred to as engineering biology) embraces the entire innovation pipeline, addressing challenges that enable the bioeconomy through the application of bio-design principles. It is a transformative platform technology offering innovative approaches for the design/redesign and fabrication of biological components and systems for biotechnology applications (1, 2). Much of the current synthetic biology activities are focused on the rapid prototyping of biosystems to deliver novel solutions to worldwide grand challenges (3). The widespread establishment of a community of biofoundries is seen as the enabler for accelerating these endeavors. Biofoundries offer a unique opportunity to harness the power of constructing biology with new process systems and automated workflows (4). At the London Biofoundry (LBF), we have focused on early-stage technology readiness levels where, through responsible innovation, we have identified new materials, sustainable products, and novel technologies that are currently being developed (5). These all have the potential to revolutionize sustainable biomanufacturing - realizing the applications of synthetic biology necessitates the continued development of enabling technologies, with the polices and praxes to ensure these are accessible to the research community.

Most biofoundries provide integrated molecular biology facilities, a core laboratory extensively automated to carry out a range of workflows and with a mantra based on the synthetic biology design-build-test-learn (DBTL) cycle. Various biofoundries are developing a complete synthetic biology technology stack to deliver complex bio-design projects that are vertically integrated. Highly efficient automated manufacturing platforms, including robotic liquid-handling equipment coupled with computer-aided design software, enable enhanced high-throughput analyses. Through such processes, biofoundries are underpinning reproducibility and enabling the quantitative precision required for modern biomanufacturing (3–5). A large scope of the bio-design space is also being exploited by machine learning (ML) and artificial intelligence (AI) approaches, whereby large-scale high-quality data as well as proper experimental design are fundamental to the leverage of ML. We are quickly recognizing that historical biological data does not always meet the requirements for ML to be effective (including a lack of standardized data measurement and annotation), so it is important that new data are collected and aggregated with these needs in mind. However, a major challenge that still exists for researchers and biofoundries is the enablement of high-throughput analytics and omics measurements, to provide the data required to feed ML and statistical learning approaches. This current bottleneck is preventing the full adoption of these approaches for bio-design and the DBTL cycle although many biofoundries are working to address this.

In summary, biofoundry infrastructure primarily provides integrated facilities for high-throughput iterative prototyping of biodesigns, prior to any scale-up such as a pilot-scale fermentation or biomanufacturing (3, 5). A notable exemplar is the Manchester Synthetic Biology Research Centre for Fine and Speciality Chemicals, where *Escherichiacoli* strains were prototyped for the production of 17 chemically diverse bio-based building blocks in 85 days from scratch (6).

Given that synthetic biology is a disruptive technology, it lends itself to commercialization via the creation of small- and medium-sized enterprises (SMEs) and early-stage start-up companies (7). As an exemplar, the LBF in conjunction with SynbiCITE (the UK Innovation and Knowledge Centre for Synthetic Biology) acts as a nucleating point for a variety of biomanufacturing applications. The LBF infrastructure has facilitated a more porous interface between industry and academia, with collaborative engagements from both sides leading to an enhanced understanding of the value each can bring to one another (see Figure 1). The LBF and other biofoundries nationally and worldwide are also leveraging upon the progressive cultural change in academia that is recognizing the value of innovation and entrepreneurial activity at a curriculum level. For example, many undergraduate students are now taught entrepreneurship modules and are actively encouraged to engage in commercialization of their ideas through multiple competitions, workshops and hackathons (8).

**Figure 1.**
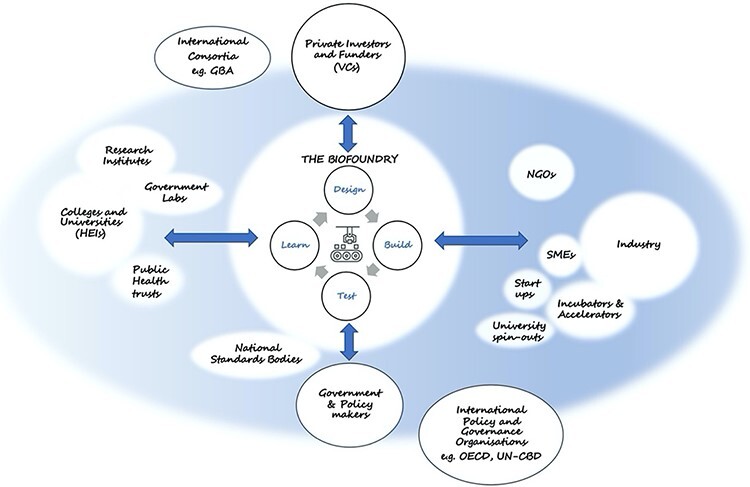
The biofoundry innovation ecosystem.

Biofoundries act at the interface of academia and industry (gray horizontal arrows) and tap into a large array of participants and resources that contribute to ongoing technology translation and innovation in synthetic biology/biotechnology. By facilitating infrastructure access to a variety of spinouts/start-ups, SMEs and industry across the synthetic biology landscape, biofoundries create value to the innovation ecosystem. Biofoundries also provide connectivity between new synthetic biology companies with venture capitalists and private investors as well as government and policy makers (gray vertical arrows). Due to the disruptive nature of synthetic biology technologies, biofoundries also provide a focus for dialogs with other stakeholders including international policy and governance organizations (e.g. Organisation for Economic Co-operation and Development (OECD) and United Nations Convention on Biodiversity (UN-CBD)) and Non-Governmental Organisations (NGOs).

## 1. Connecting academia with industry

Public-funded biofoundries are generally open-access and sit at the interface between academia and industry. A distinct feature of biofoundries is that they can be enablers for many spin-outs and start-up companies, by providing access to expensive infrastructure. Biofoundries can enable new companies to accelerate the development of new products without reinventing the wheel every time. They can offer access to sophisticated computational design tools, novel genetic parts, hardware and software infrastructure, bespoke workflows and assay development, in addition to expertise and ‘know-how’ to help address-specific biotechnology research challenges. Biofoundries can also provide these tools to the academic scientific community, with easy access to the skills and technologies required to accelerate synthetic biology academic research. As such, they are janus facing, working with both academia and industry, and ideally positioned to accelerate commercialization and translation of synthetic biology technologies and applications worldwide (Figure 1; 3, 7).

The LBF is purposefully embedded within a hub for innovation and entrepreneurship (the Imperial Translation and Innovation Hub) with easy access to several incubators and start-up companies (9, 10). Purposeful infrastructure integration is commonplace, another example being the Earlham Biofoundry (11). Located within the Norwich Research Park, the Earlham Institute has access to a thriving community of businesses, researchers and clinicians. Many of the early-stage companies are working on a wide variety of biotechnology projects, often emerging as university start-ups that are encountering the financial and logistical realities of running a technology company. Biofoundry infrastructure allows many of these micro-companies to rapidly evaluate their new ideas and biotechnology designs and obtain robust experimental data to de-risk uncertainty in their investment process (Figure 1). Our own experiences of working with companies, ranging from early-stage start-ups to large tier 1 corporations and pharmaceutical companies, have allowed the development of new workflows, which can be reused to support proof-of-concept and larger scale R&D projects. Many public-funded biofoundries, including the LBF, Earlham Agile, and Edinburgh Genome Foundry (EGF), market their technical services as well as educational tools such as workshops, conferences, training courses and webinars. These activities have assisted many companies to determine how biofoundry infrastructure can facilitate their own innovation and R&D pipelines. The US-based Agile Biofoundry is a distributed consortium of national labs and academic intuitions dedicated to accelerating biomanufacturing and the bioeconomy through working closely with industry (12). Similarly, in Singapore, the SynCTI biofoundry has established major flagship research programs and industrial network focused on accelerating innovative biosolutions (13).

As shown in Figure 1, not only can biofoundries provide infrastructure access for start-ups, SMEs, larger companies and academic researchers, they can also coordinate and contribute to activities around government policy, governance and standards. International consortia of biofoundries, like the GBA, also provide a wide network for knowledge exchange and training. Importantly, biofoundries can also provide a gateway for public dialog on the uses and applications of synthetic biology technology.

Liaison with industry by biofoundries is helping to create a framework for identifying and monitoring standardization requirements for new synthetic biology tools, technologies and applications. Biofoundries are able to systemize an evolving list of priorities that are unique to synthetic biology, including simple biological parts, the definition and adoption of new chassis, data standards and the development reference materials to support the metrology of gene expression flow and also adoption costs and techno-economic analysis (TEA) (14). Through the use of biofoundries, the field of synthetic biology is increasingly engaged with the scientific, technical, operational and semantic standards required for the field to become a full-fledged engineering discipline. These developments in standards will both enable and accelerate the industrial translation of synthetic biology, thus allowing more reproducible processes governed by over-arching regulatory requirements (15, 16).

## 2. Standards and metrology agenda

As an exemplar, the LBF is actively engaged in an Industrial Strategy Challenge Fund (ISCF) project with the National Physical Laboratory (NPL) through the Virtual Metrology and Standards Centre for Engineering Biology that was established in 2018 (17). A high-throughput cell-free platform for intracellular measurements in miniaturized format is currently being developed alongside NPL biometrology scientists, with whom we are also generating candidate reference materials and biosynthesis protocols (18–20). Through a series of workshops, we are also exploring a number of key technical, regulatory and societal challenges to enable the development and adoption of standards in synthetic biology, as well as how these could be implemented by our industrial stakeholders (14, 15).

Through a more extensive industrial engagement by the broader biofoundry community, automation equipment and easy to adopt workflows have a clear role to play in addressing early-stage biotechnology/synthetic biology projects. Start-up companies and researchers interested in starting a company need easy access to automation infrastructure early on in their growth; however, they also need advice on scale-up bioprocesses and access to TEA expertise, as part of investor due diligence. These areas are not widely available in public-funded biofoundries, and these gaps need to be filled quickly. One exception is the US Agile Biofoundry, where the economic feasibility of new biotechnology processes can be assessed as part of their infrastructure offering (12). A widespread use of biofoundries for strain engineering, involving genetic design and DNA construction, is also allowing improved standardization for these foundational technologies (21, 22).

We view biofoundries as a key to developing and promoting standards and reference materials necessary to deliver the promise of synthetic biology, both in terms of systematic design and engineering biological systems. The unique combination of high-throughput infrastructure and technical expertise in molecular biology, analytics, automation, engineering and software development provides an excellent, integrated and agile capability to quickly establish platforms for prototyping biological measurement standards, interoperability and developing new liquid-handling workflows. The Global Biofoundry Alliance (GBA) is vesting in standards setting through its metrology, reproducibility and data quality working group activities (23).

## 3. International consortia and encouraging reproducibility

The GBA was launched at an event at the University of Kobe, Japan, in May 2019. The GBA is an impressive network, already comprising over 30 international biofoundries, that brings together the world’s leading non-commercial public-funded biofoundries. By sharing knowledge, infrastructure and expertise, the GBA is playing a key role accelerating the capabilities of biofoundries in exploring globally relevant and societally impactful grand challenges as well as metrology, standards setting, interoperability and software developments (4, 23).

‘Global Challenges’ is an area of activity within the GBA that is particularly relevant to the current pandemic, where experiences and biofoundry technology developments around COVID-19 have been shared. The GBA also provides an international forum to build a community of biofoundry practitioners and users (4, 23). GBA biofoundries operate in an open technology environment that facilitates the development of common standards, and open-source reference materials, and tools across the global community. We see these, and the associated protocols and guidelines, as playing an invaluable role in accelerating the application of synthetic biology technologies worldwide. One area of intense activity in the GBA, and more broadly in the synthetic biology field, encompasses software and data standards. Data standards enable the ready exchange of information from the synthetic biology workflow, allowing repositories and tools to be connected from a diversity of sources (7, 16). A key area of development is SBOL, or the ‘Synthetic Biology Open Language’. SBOL is an open standard for the representation of in silico biological design and standardizes data used by synthetic biology practitioners—from users to software developers to wet-lab biologists (24).

The associated protocols and guidelines that are affiliated with common standards, data management and reference materials across the global community, are having a valuable role in shifting the mindset of young entrepreneurs. These ‘soft standards’ facilitate help the routine translation of synthetic biology, although the industry assumption that standards may limit flexibility but increase interoperability is also relevant. That said, we see good standards as increasing creativity and flexibility, because translational research objectives will be within closer reach for many new start-up companies (7, 16).

As an exemplar, the LBF and larger synthetic biology community at Imperial College recently established a collaboration with the US company, Riffyn, to modernize data management. Riffyn has developed a computer-aided design approach to biotechnology research and development that breaks down data silos to deliver clean, contextualized and connected data for real-time analysis. This open-science collaboration is allowing Riffyn’s cloud-based process data system to be utilized in a biofoundry setting. We hope the outputs of this collaboration will encourage increasing reproducibility across the broader synthetic biology community, providing us with a consistent and standardized way of sharing data both locally and externally. Having the means to implement industrially proven data systems, like Riffyn, has given us the capacity to improve the repeatability of synthetic biology research and development (25).

## 4. Repurposing synthetic biology technology to address global challenges

The success of our biofoundry to date has been, in part, due to its academic and public sector partnerships, and involvement in other consortia that serve the broader bioeconomy (26). These recently include important collaborative projects focused on SARS-CoV-2 testing within the Department of Infectious Disease at Imperial, the National Health Service (NHS) Imperial Trust, the UK Dementia Research Institute and a pan-London NHS diagnostic laboratory network. By demonstrating that our biofoundry infrastructure could be rapidly re-purposed and our platforms recalibrated to overcome supply chain issues, we were able to highlight the flexible and inter-operable nature of biofoundry platform technology in a ‘real-world’ setting. The LBF’s workflow and open platforms are now in use in NHS pathology labs for frontline automated diagnostic testing in the UK and in other countries providing >250 000 COVID-19 tests (27). This partnership is a good example of how biofoundries can help health services around the world develop low-cost reagent-agnostic open testing platforms for other infectious diseases. The LBF has extended its COVID response to include new workflows for sample pooling that can be implemented in frontline testing, as well as High-Throughput (HTP) Sequencing of SARS-CoV-2 viral variants (28). Another notable example is the rapid establishment of a high-throughput Clinical COVID-19 Testing Service Bundle at the DAMP Lab, increasing testing capacity to the Boston University’s community (29). These exemplars illustrate the agility of biofoundries to repurpose their existing workflows for specific applications, which is one of the major strengths and unique features of biofoundries.

## 5. Sustainability and creating value

Biofoundries are in a unique position to broker and manage interactions between stakeholders. In publicly funded business-led research partnerships, biofoundries are able to accelerate technology readiness levels of academic research programs and deliver economic, social and cultural prosperity. However, to enable such partnerships, biofoundries also need to be sustainable. In our experience, the key to medium- and long-term sustainability is the creation of a core client base of academics and industry. This can be via strategic partnerships combined with a flexible ‘fee-for-service’ model to deliver a variety of proof-of-concept projects or collaborative research grants where costs for accessing the biofoundry infrastructure are included. Evaluation of the potential client base should extend beyond the host institute’s ecosystem and embrace the broader national and international research communities. While these strategies require significant community engagement, they can also lead to the identification of additional funding sources and alternative funding models. For example, in the UK, the Innovate UK Smart Grant scheme, EPSRC Prosperity Partnerships and direct Venture Capital injection (30). A collaboration led by the University of Edinburgh and the Edinburgh Genome Foundry and FUJIFIM Diosynth Biotechnologies UK (FDB) has recently won £8.7 million Prosperity Partnership funding to develop cost-effective ways to manufacture modern antibody-based medicines (31).

In the UK, smaller companies tend to organize into sector-specific clusters to enable business-to-business transactions and workforce mobility (9–11). By being geographically and economically accessible to such clusters or indeed facilitating the formation of them, biofoundries can play a specific key role in the development of metrology infrastructure to accelerate industrial adoption of new biotechnologies and applications (5, 11, 12, 23). Biofoundries can also play a crucial role in coordinating the efforts of academia, government and industry (Figure 1), through focused meetings that foster interdisciplinary collaborations around shared objectives and promoting new technology developments to a wider group of stakeholders (7, 14, 17). As part of this, we would like to see increased interoperability, coordination of labor, reproducibility and reuse of people’s efforts. We envisage biofoundries as being central to nucleating start-ups and SME clusters, and drawing in the consensus of private and public companies, universities and policymakers. This in turn will facilitate the implementation of reliable, robust and affordable standardization of synthetic biology technologies that has been endorsed by a broad spectrum of synthetic biology users. Local governments and regional funding bodies could support such platform technologies within a biofoundry hub, to both de-risk private investment in new start-ups, while also creating an active ecosystem of new companies around synthetic biology technology developments.

## 6. Future outlook

Biofoundries reflect the infrastructure required to address the data-rich era that contemporary biotechnology encapsulates, both politically and technologically. The UK biofoundry network had been instrumental in engaging with policymakers, who are helping realize that a new era of bio-based production is required to drive their bioeconomies (32). Increased public investment for synthetic biology research is also required, as well as further investment and upgrades for biofoundries, including staff development and retention. As industrial processes progress, we will need to implement new infrastructure to supersede the existing synthetic biology technology stack to deliver on projects of increasing complexity and which address future challenges. The current pandemic has accentuated some of the weak links in biosecurity preparedness and the need to develop workflows on automation platforms that can be readily redeployed in diagnostic laboratories (27–29). It would be wise to further establish automated high-throughput infrastructures, centered around a community of practice, involving academics and industrialists as well as wider stakeholders, to enable the rapid transfer and benchmarking of biosecurity protocols on new platforms, including distributed and accelerated vaccine developments.

Biofoundries of the future may not only be able to parallelize, automate and miniaturize the steps in the synthetic biology design-build-test cycles but also autonomously learn about the design and construction of biosystems. AI and ML may narrow synthetic biology’s potential design solutions to a number that can be efficiently generated and tested at scale. With the growth of AI and deep learning, it is plausible that design rules for constructing biological systems in specific host cells for different applications will be developed (33). In strain engineering, design rules could also consider downstream scale-up processes accelerating biomanufacturing applications. This would then allow scientists to focus on conceptual innovations, with their ideas implemented rapidly in a biofoundry.

The current climate has further highlighted a future, whereby synthetic biology industrialists could use cloud computing to access more centralized biofoundries to carry out more complex experimental workflows. We envisage a seamless interface between cloud-based upstream prototyping activities within our biofoundry community and downstream industrial biomanufacturing. In this scenario, biofoundries can be considered as a joint infrastructure investment, where many research institutions and other stakeholder can combine their skills and funding assets.

## References

[1] Engineering Biology Research Consortium . https://ebrc.org/ (25 April 2021, date last accessed).

[2] HM Government . (2018) Growing the Bioeconomy. https://assets.publishing.service.gov.uk/government/uploads/system/uploads/attachment_data/file/761856/181205_BEIS_Growing_the_Bioeconomy__Web_SP_.pdf (25 April 2021, date last accessed).

[3] Freemont P.S. (2019) Synthetic biology industry: data-driven design is creating new opportunities in biotechnology. *Emerg. Top Life Sci.*, 3, 651–657. 33523172 10.1042/ETLS20190040 PMC7289019

[4] Hillson N. , Caddick M., Cai Y., Carrasco J.A., Chang M.W., Curach N.C., Bell D.J., Feuvre R.L., Friedman D.C., Fu X. et al. (2019) Building a global alliance of biofoundries. *Nat. Commun.*, 10. 10.1038/s41467-019-10079-2 PMC6506534 31068573

[5] The London Biofoundry . https://www.londonbiofoundry.org (25 April 2021, date last accessed).

[6] Robinson J.C. , Carbonell P., Jervis A.J., Yan C., Hollywood C.A., Dunstan M.S., Currin A., Swainston N., Spiess R., Taylor S. et al. (2020) Rapid prototyping of microbial production strains for the biomanufacture of potential materials monomers. *Metab. Eng.*, 60, 168–182. 32335188 10.1016/j.ymben.2020.04.008 PMC7225752

[7] Clarke L. and Kitney R. (2020) Developing synthetic biology for industrial biotechnology applications. *Biochem. Soc. Trans.*, 48, 113–122. 32077472 10.1042/BST20190349 PMC7054743

[8] International Genetically Engineered Machine . https://igem.org (25 April 2021, date last accessed).

[9] Imperial ThinkSpace . https://www.imperial.ac.uk/thinkspace/i-hub/ (25 April 2021, date last accessed).

[10] Open Cell Labs . https://www.opencell.bio (25 April 2021, date last accessed).

[11] Earlham Biofoundry . https://www.earlham.ac.uk/earlham-biofoundry (25 April 2021, date last accessed).

[12] Agile BioFoundry . https://agilebiofoundry.org (25 April 2021, date last accessed).

[13] SynCTI . https://syncti.org (25 April 2021, date last accessed).

[14] Fostering a Synthetic Biology Standards Agenda through the Centre for Engineering Biology, Metrology and Standards . http://www.synbicite.com/news-events/synbicite-blog/fostering-synthetic-biology-standards-agenda-throu/ (25 April 2021, date last accessed).

[15] Beal J. , Goñi-Moreno A., Myers C., Hecht A., de Vicente M.D.C., Parco M., Schmidt M., Timmis K., Baldwin G., Friedrichs S. et al. (2020) The long journey towards standards for engineering biosystems: are the molecular biology and the biotech communities ready to standardise? *EMBO Rep.*, 21, e50521. 10.15252/embr.202050521 PMC7202200 32337821

[16] Beal J. , Haddock-Angelli T., Farny N. and Rettberg R. (2018) Time to get serious about measurement in synthetic biology. *Trends Biotechnol.*, 36, 869–871. 29880229 10.1016/j.tibtech.2018.05.003

[17] The UK Centre for Engineering Biology, Metrology and Standards . https://www.npl.co.uk/projects/centre-engineering-metrology (25 April 2021, date last accessed).

[18] Vila-Gómez P. , Noble J.E. and Ryadnov M.G. (2021) Peptide nanoparticles for gene packaging and intracellular delivery. *Methods Mol. Biol.*, 2208, 33–48. 32856254 10.1007/978-1-0716-0928-6_3

[19] De Santis E. and Rydanov M.G. (2021) Imaging and 3D reconstruction of de novo peptide capsids. *Methods Mol. Bio.*, 2208, 149–165. 32856261 10.1007/978-1-0716-0928-6_10

[20] Versailles Project on Advanced Materials and Standards (VAMAS) . http://www.vamas.org (25 April 2021, date last accessed).

[21] Myers C.J. , Beal J., Gorochowski T.E., Kuwahara H., Madsen C., McLaughlin J.A., Misirli G., Nguyen T., Oberortner E., Samineni M. et al. (2017) A standard-enabled workflow for synthetic biology. *Biochem. Soc. Trans.*, 45, 793–803. 28620041 10.1042/BST20160347

[22] Zhang J. , Yongcan C., Lihao F., Erpeng G., Bo W., Lei D. and Tong S. (2021) Accelerating strain engineering in biofuel research via build and test automation of synthetic biology. *Curr. Opin. Biotechnol.*, 67, 88–98. 33508635 10.1016/j.copbio.2021.01.010

[23] Global Biofoundries Alliance . https://biofoundries.org (25 April 2021, date last accessed).

[24] Baig H. , Fontanarrosa P., Kulkarni V., McLaughlin J.A., Vaidyanathan P., Bartley B., Beal J., Crowther M., Gorochowski T.E., Grünberg R. et al. (2020) Synthetic biology open language (SBOL) version 3.0.0. *J. Integr. Bioinform.*, 17. 10.1515/jib-2020-0017 PMC7756618 32589605

[25] New Riffyn-Imperial partnership set to accelerate synthetic biology research . https://www.imperial.ac.uk/news/205254/new-riffyn-imperialpartnership-accelerate-synthetic-biology/ (25 April 2021, date last accessed).

[26] BioRoboost . https://standardsinsynbio.eu (25 April 2021, date last accessed).

[27] Crone M.A. , Priestman M., Ciechonska M., Jensen K., Sharp D.J., Anand A., Randell P., Storch M. and Freemont P.S. (2020) A role for Biofoundries in rapid development and validation of automated SARS-CoV-2 clinical diagnostics. *Nat. Commun.*, 11, 4793. 10.1038/s41467-020-18130-3 PMC7479142 32900994

[28] Crone M.A. , Randell P., Herm Z., Missaghian-Cully S., Perelman L., Pantelidis P. and Freemont P.S. (2021) Design and implementation of an adaptive pooling workflow for SARS-CoV-2 testing in an NHS diagnostic laboratory. *SSRN*. https://papers.ssrn.com/sol3/papers.cfm?abstract_id=3799293. 10.12688/wellcomeopenres.17226.1 PMC8591522 34796279

[29] DAMP Lab . https://www.damplab.org/covid-19 (25 April 2021, date last accessed).

[30] Innovate UK Smart Grants . https://apply-for-innovation-funding.service.gov.uk/competition/810/overview (25 April 2021, date last accessed).

[31] Pre-announcement: Business and Academia Prosperity Partnership . https://www.ukri.org/opportunity/business-and-academia-prosperity-partnership/ (25 April 2021, date last accessed).

[32] (2020) *Jumpstarting the**Recovery*. *N**avigating the**Return**Across**Industries*. McKinsey Publishing, New York NY.

[33] Radivojević T. , Costello Z., Workman K. and Hector Garcia M. (2020) A machine learning automated recommendation tool for synthetic biology. *Nat. Commun.*, 11, 4879. 10.1038/s41467-020-18008-4 PMC7519645 32978379

